# Frabin and other related Cdc42-specific guanine nucleotide exchange factors couple the actin cytoskeleton with the plasma membrane

**DOI:** 10.1111/j.1582-4934.2008.00345.x

**Published:** 2008-04-10

**Authors:** Hiroyuki Nakanishi, Yoshimi Takai

**Affiliations:** a Department of Molecular Pharmacology, Graduate School of Medical Sciences, Kumamoto UniversityKumamoto, Japan; b Department of Molecular Biology and Biochemistry, Osaka University Graduate School of Medicine/Faculty of MedicineSuita, Japan; c Division of Molecular and Cellular Biology, Department of Biochemistry and Molecular Biology, Kobe University Graduate School of MedicineKobe, Japan

**Keywords:** frabin, FGD1, guanine nucleotide exchange factor, Cdc42, Rac, actin cytoskeleton, plasma membrane, filopodia, lamellipodia

## Abstract

Frabin, together with, at least, FGD1, FGD2, FGD3 and FGD1-related Cdc42-GEF (FRG), is a member of a family of Cdc42-specific gua-nine nucleotide exchange factors (GEFs). These proteins have multiple phosphoinositide-binding domains, including two pleckstrin homology (PH) domains and an FYVE or FERM domain. It is likely that they couple the actin cytoskeleton with the plasma membrane. Frabin associates with a specific actin structure(s) and induces the direct activation of Cdc42 in the vicinity of this structure(s), resulting in actin reorganization. Furthermore, frabin associates with a specific membrane structure(s) and induces the indirect activation of Rac in the vicinity of this structure(s), resulting in the reorganization of the actin cytoskeleton. This reorganization of the actin cytoskeleton induces cell shape changes such as the formation of filopodia and lamellipodia.

- Introduction
- Molecular structures of frabin and other related GEFs
- Tissue distribution, subcellular localization and splicing variants of frabin and other related GEFs
- Cellular activities of frabin and other related GEFs
- Roles of the domains of frabin in cellular activities
- Mode of action of frabin in cell shape changes
- Mode of activation of frabin and FRG
- Involvement of *FGD4*/*frabin* and *FGD1* in human diseases
- Conclusions and perspectives

## Introduction

Rho family small GTP-binding proteins (G proteins), including Cdc42, Rac and Rho, regulate the actin cytoskeleton-dependent cellular activities, including cell shape changes, cell migration, cell adhesion and cytokinesis [1, 2]. These Rho family small G proteins also regulate other cellular activities such as the c-Jun N-terminal kinase (JNK) and p38 mitogen-activated protein kinase cascade, an NADPH oxidase enzyme complex and the transcription factor NF-κB [2]. Of the actin cytoskeleton-dependent cellular activities in fibroblasts, such as NIH 3T3 and Swiss 3T3 cells, Cdc42 regulates the formation of filopodia, Rac regulates the formation of lamellipodia and ruffles and Rho regulates the formation of stress fibers and focal adhesions [1, 2]. Cdc42 and Rac activate the Arp2/3 complex through their respective target proteins, Wiskott-Aldrich syndrome protein (WASP)/neural (N-)WASP and WASP-family verprolin-homologous protein (WAVE) [3]. The Arp2/3 complex interacts with the sides of the pre-existing actin filaments (F-actin) to promote actin polymerization and generate a branched F-actin network. Rho promotes actin polymerization through two distinct targets, p160 and mDia [2]. Despite both Cdc42 and Rac activating the Arp2/3 complex, it is unclear how they induce the formation of morphologically distinct structures, for example, filopodia with straight bundles of F-actin and lamellipodia with branched F-actin. Recent evidence indicates that Cdc42 stimulates actin polymerization through another target protein, mDia2, leading to the formation of filopodia [4].

Like other G proteins, the Rho family members have two interconvertible forms: GDP-bound inactive and GTP-bound active forms [1, 2, 5]. Their interconversion is tightly controlled by three types of regulators: guanine nucleotide exchange factors (GEFs) that stimulate the conversion from the GDP-bound form to the GTP-bound form, GDP dissociation inhibitors (GDIs) that inhibit this reaction and GTPase-activating proteins (GAPs) that stimulate the conversion from the GTP-bound form to the GDP-bound form. The modes of action and activation of the Rho family members by these regulators are proposed to be as follows [1, 5]: in the cytosol, the small G proteins are complexed with GDIs and maintained in the GDP-bound inactive form. The GDP-bound form is first released from GDIs by a still unknown mechanism and is converted to the GTP-bound form by the action of GEFs. The GTP-bound form then interacts with downstream effectors. Thereafter, the GTP-bound form is converted to the GDP-bound form by the action of GAPs. The GDP-bound form then forms a complex with GDIs and returns to the cytosol.

GEFs for the Rho family members share two conserved domains: a Dbl homology (DH) domain, for which the Dbl oncogene product is the prototype, and a pleckstrin homology (PH) domain adjacent the DH domain [1, 2]. Recently, the members of a newly discovered family have been shown to serve as GEFs: CDM proteins, including Ced-5, Dock180 and Myoblast city, act as Rac-specific GEFs; and zizimin proteins act as Cdc42-specific GEFs [6]. Thus, the number of GEFs for the Rho family members is growing; it is important to clarify how each GEF is activated and how each GEF activates the Rho family G proteins. These studies will lead to an understanding of the mechanisms that underlie the spatial and temporal activation of the Rho family small G proteins within cells in response to external or internal stimuli.

Many GEFs for the Rho family members were originally identified as oncogenes [1, 2]; in contrast, *FGD1*, which contains DH and PH domains, was discovered by positional cloning as the gene responsible for faciogenital dysplasia (FGDY) [7]. FGD1 shows specific GEF activity towards Cdc42 and induces the formation of filopodia and the activation of JNK through the activation of Cdc42 [8]. Subsequently, *FGD2* and *FGD3* were identified by genetic searches as *FGD1* homologues [9, 10]. We isolated frabin, a Cdc42-specific GEF, as an F-actin-binding (FAB) protein showing significant homology to FGD1 [11]. Like FGD1, frabin induces the formation of filopodia through the activation of Cdc42 in fibroblasts [11–13]. Here, we review frabin and other related Cdc42-specific GEFs.

## Molecular structures of frabin and other related GEFs

**Fig. 1:**
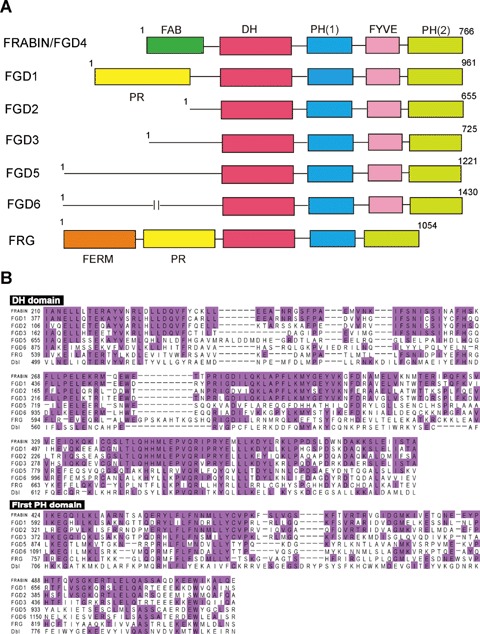
Molecular structures of frabin and other related GEFs. (**A**) Domain organization. PH(1), first PH domain; PH(2), second PH domain; PR, proline-rich domain. **(B)** Sequence comparison of DH and first PH domains. Colored boxes, identical residues.

Frabin, FGD1 [7], FGD2 [9], FGD3 [10], FGD5, FGD6 and FRG (FGD1-related Cdc42-GEF) possess a similar domain organization (Fig. 1A). FGD5 and FGD6 have recently been deposited in the database as FGD1 homologues. FRG was originally identified by genetic searches to be a protein containing sequence homology with the DH domain of FGD1 [14]. Frabin consists of an FAB domain, a DH domain, a first PH domain adjacent to the DH domain, an FYVE domain and a second PH domain, in order, from the N-terminus to the C-terminus [11]. The amino acid sequence of the FAB domain shows no significant homology to those of FGDs; each FGD has a unique N-terminal region. The amino acid sequence following the FAB domain is highly homologous to those of FGDs (Fig. 1B). Frabin binds along the sides of F-actin through the FAB domain [11]. Full-length frabin causes F-actin to associate into bundles, while the FAB domain alone does not show bundling activity. It is likely that frabin forms an oligomer with multiple FAB domains, and thereby shows F-actin-bundling activity. The N-terminal pro-line-rich domain of FGD1 has been shown to bind to F-actin through its interaction with the FAB proteins, cortactin and mAbp1 [15]. It is possible that the N-terminal regions of other FGDs also directly or indirectly interact with F-actin.

DH domains encode a principal GEF catalytic unit and are required to stimulate GDP release from the Rho family small G proteins [1, 2]. PH domains are found in many molecules and are known for their ability to target cellular membranes by binding to phosphoinositides [16]. PH domains adjacent to the DH domains of several GEFs have been shown to bind specifically to nucleotide-free small G proteins and assist effective guanine nucleotide exchange reactions [17]. Disruption of these interactions between the PH domains and small G proteins by site-directed mutagenesis inhibits GEF activities [18]. Thus, PH domains adjacent to the DH domains not only serve in directing subcellular localization, but also participate in the binding of small G proteins to facilitate guanine nucleotide exchange reactions. The amino acid sequence of the DH domain and the first PH domain of frabin shows significant homology to those of FGD1, FGD2, FGD3, FGD5 and FGD6 (Fig. 1B). A fragment of frabin containing the DH domain and the first PH domain stimulates the guanine nucleotide exchange reaction of Cdc42, but not that of Rho or Rac, in a cell-free assay system [12]. Rho GDI inhibits the GEF activity of frabin on Cdc42. A similar fragment of FGD1 also shows Cdc42-specific GEF activity [8]. The GEF activities of FGD2, FGD3, FGD5 and FGD6 have not been reported, but the sequence similarities suggest that they also function as Cdc42-specific GEFs.

FYVE domains have been found in many proteins to be involved in membrane trafficking and phosphoinositide metabolism and have been shown to specifically interact with phos-phatidylinositol 3-phosphate (PI3P) [19]. A large population of PI3P is found in the early endosomes, multi-vesicular bodies and phagosomes. A small population of PI3P is identified in the nucleus, mitochondria, Golgi and plasma membrane. FYVE domains are principally defined by three conserved sequences: the N-terminal WxxD, the central RR/KHHCR and the C-terminal RVC motifs that form a compact PI3P-binding site, but both frabin and FGD1 possess atypical FYVE domains that lack an N-terminal WxxD motif [18]. FGD1 FYVE domain was experimentally shown to recognize both PI3P and phosphatidylinositol 5-phosphate [20]. Thus, frabin and FGDs have not only two PH domains, but also an FYVE domain, and this domain organization suggest that they serve as cross-linkers between the membranes and the actin cytoskeleton.

The amino acid sequence of the DH domain of FRG shows significant homology to those of FGD1 and frabin (Fig. 1B). Consistently, FRG shows Cdc42-specific GEF activity in a cell-free assay system [14]. FRG consists of an FERM domain, a proline-rich domain, a DH domain and two PH domains, in order, from the N-terminus to the C-terminus. FERM domains, also called band 4.1 homology domains, were initially identified in the peripheral membrane proteins that function as cross-linkers between the plasma membrane and F-actin [21, 22]. FERM domains bind to both phosphoinositides and the cytoplasmic tails of integral membrane proteins. FRG may also serve as a Cdc42-specific GEF that couples the plasma membrane with the actin cytoskeleton.

## Tissue distribution, subcellular localization and splicing variants of frabin and other related GEFs

*Frabin* is expressed in all rat tissues, including the heart, brain, spleen, lung, liver, skeletal muscle, kidney and testis [12]. In cultured rat hippocampal neurons, frabin is highly concentrated at filopodia in the growth cones. Mouse frabin has two smaller splicing variants [23]. The original biggest, middle and smallest variants are named frabin-α, -β and -γ, respectively. In this article, unless otherwise indicated, frabin represents the α form. Frabin-β lacks the second PH domain, whereas frabin-γ lacks the FYVE domain and the second PH domain. These variants are expressed in all tissues, but their expression levels differ among tissues. The three different splicing variants of frabin induce partly different morphological changes. Thus, frabin-α, -β and -γ may have different physiological functions. Human FGD4/frabin has recently been shown to have other splicing variants, one of which is deprived of the FAB domain [24]. This splicing variant may also have a different function.

FGD1, FGD2 and FGD3 are mainly expressed in restricted tissues, particularly in the bone, spleen and skeletal muscle, respectively [7, 9, 10, 25]. FGD1 is localized at the subcortical actin cytoskeleton and the Golgi membrane in the osteoblast-like cell line, MC3T3-E1 [26]. It has been shown that Cdc42 and N-WASP are localized together, in part, at the Golgi membrane and that they regulate vesicle trafficking from the Golgi to the ER [4]. FGD1 may be involved in this Golgi–ER transport. FGD1 has also two splicing variants that only possess a proline-rich domain and an incomplete DH domain [27, 28]. These splicing variants may regulate the activation of Cdc42 by competing with the longest type of FGD1. FRG is ubiquitously expressed and localized to cell–cell adhesion sites [14, 29], but splicing variants have not been reported.

## Cellular activities of frabin and other related GEFs

**Fig. 2:**
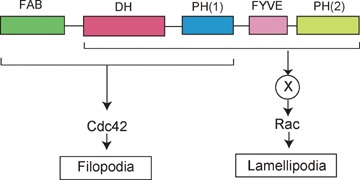
Roles of the domains of frabin in cell shape changes. The N-terminal region of frabin containing the FAB domain, the DH domain and the first PH domain induces the formation of filopodia through the direct activation of Cdc42. The C-terminal region of frabin containing the DH domain, the first PH domain, the FYVE domain and the second PH domain induces the formation of lamellipodia through the indirect activation of Rac.

Expression of the full-length frabin induces the formation of filopodia and the activation of JNK through the activation of Cdc42 in fibroblasts [11–13] (Fig. 2). Furthermore, the expression of frabin induces the formation of not only filopodia but also lamellipodia in fibroblasts [13]. This morphological change is inhibited by a dominant-negative mutant (DN) of Rac, indicating that the formation of lamellipodia is mediated by the activation of Rac. Frabin does not show GEF activity towards Rac, indicating that the activation of Rac is an indirect action. In epithelial cells, such as MDCK cells, frabin induces the formation of microspikes at the basal area of the lateral membrane through the activation of both Cdc42 and Rac, although a constitutively active mutant (CA) of Cdc42 or Rac alone, or both, does not induce the formation of microspikes [29]. These different effects between frabin and the small G protein CAs in MDCK cells suggest that frabin has an activity(s) other than the activation of the small G proteins, as described below. Thus, fra-bin has three-typed cellular activities: *(i)* Cdc42-dependent, *(ii)* Rac-dependent and (iii) Cdc42/Rac-independent.

Like frabin, FGD1 and FGD3 exhibit Cdc42-dependent cellular activities: they induce the formation of filopodia and the activation of JNK in fibroblasts [8, 10, 30]. However, it is not clear whether FGD1 and FGD3 induce the Rac-dependent formation of lamellipo-dia. FRG has also been shown to exhibit Cdc42-dependent cellular activities: it induces the activation of JNK in 293T epithelial cells [14] and regulates nectin- and cadherin-based cell–cell adhesion and cell polarity in MDCK epithelial cells [31, 32]. FRG also induces the Cdc42-dependent formation of filopodia and spines in hippocampal neurons [33].

## Roles of the domains of frabin in cellular activities

A fragment of frabin containing the DH domain and the first PH domain shows Cdc42-specific GEF activity in a cell-free assay system, but this fragment does not induce the formation of filopodia or lamellipodia or the activation of JNK in fibroblasts [11–13]. For the formation of filopodia, the FAB domain is additionally required, suggesting that the association of frabin with the actin cytoskeleton is necessary for this activity (Fig. 2). A fragment containing the FAB domain is recruited to Cdc42 CA-formed filopodia, but not to Rho CA-formed stress fibers [34]. Furthermore, co-expression of this domain inhibits the formation of filopodia induced by the full-length frabin. These data suggest that the FAB domain competes with the full-length frabin for the association with a specific actin structure(s), and thereby inhibits the formation of filopodia. Because actin structures are determined on the basis of the various FAB proteins they are composed of, it is possible that the association of the FAB domain with the specific actin structures is mediated by interactions not only with F-actin itself, but also with a specific FAB protein(s).

As described above, the expression of Cdc42 CA or Rac CA, or both, does not result in the generation of microspikes in MDCK cells [29]. However, the microspikes are formed when Cdc42 CA is co-expressed with a fragment of frabin minimally including the FAB domain and a mutated DH domain that lacks Cdc42-activating activity [35]. These data suggest that the region containing the FAB and DH domains directly reorganizes the actin cytoskeleton in a Cdc42-independent manner, and that both the Cdc42-activating and F-actin-modulating activities of frabin are required for the generation of microspikes in MDCK cells. Consistently, FGD1 also promotes Cdc-42-independent actin polymerization through its interaction with cortactin [15, 36]. Cortactin is a c-Src substrate that binds to and activates the Arp2/3 complex, leading to the formation of a branched F-actin network [37, 38]. This activity of cortactin has been shown to be enhanced by its interaction with FGD1 [36].

On the other hand, the FYVE domain and the second PH domain, in addition to the DH domain and the first PH domain, are necessary for the formation of lamellipodia and the activation of JNK, suggesting that the association of frabin with membranes is required for these activities [12, 13] (Fig. 2). Thus, different domains of frabin are involved in distinct morphological changes through the activation of Cdc42 and Rac. A fragment of frabin containing the FYVE domain is recruited to Rac CA-induced membrane ruffles [34]. The expression of the fragment containing the mutated DH, first PH, FYVE and second PH domains inhibits the formation of membrane ruffles induced by the full-length frabin; however, the expression of shorter fragments, such as the FYVE domain alone, does not result in this inhibitory action. It is likely that this fragment, containing the DH, first PH, FYVE and second PH domains, competes with the full-length frabin for the association with a specific membrane structure(s). The highly ordered structure of this fragment may be required for its interaction with the specific membrane structure(s) because shorter fragments, such as the FYVE domain alone, do not show a DN effect. It has recently been shown that phosphoinositide-binding domains, such as BAR, EFC and RCB/IMD domains, have profound effects on the membranes with which they interact [3]. These domains have been shown to sense and/or generate membrane curvature for membrane invagination during endocytosis. The region of fra-bin containing the DH, first PH, FYVE and second PH domains may also sense and generate a specific membrane structure (curvature) for inducing cell shape changes.

## Mode of action of frabin in cell shape changes

**Fig. 3:**
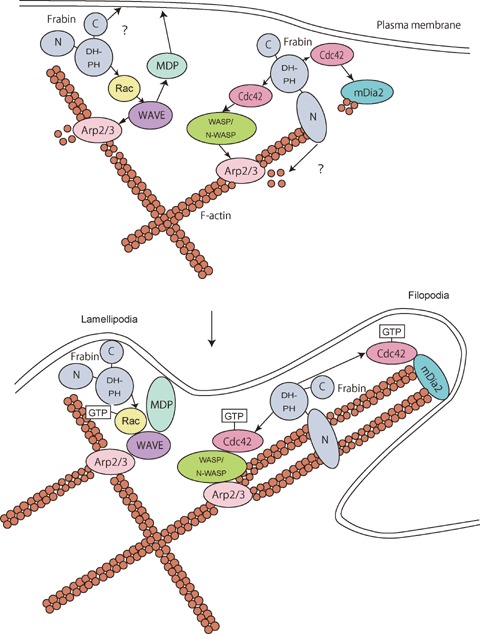
Model for the mode of action of frabin in cell shape changes. Initially, frabin is recruited to a pre-existing specific actin structure(s) through the N-terminal region, where frabin directly reorganizes the actin cytoskeleton in a Cdc42-independent manner. In addition, frabin activates Cdc42 through the DH and first PH domains in the vicinity of the actin structure(s), resulting in the WASP/N-WASP-Arp2/3 system-induced generation of branched F-actin. Frabin causes F-actin to associate into bundles. Furthermore, Cdc42 activated by frabin stimulates actin polymerization through mDia2. The co-operation of Cdc42-independent and Cdc42-dependent actin reorganization finally induces the formation of filopodia. Frabin is also recruited to a specific membrane structure(s) through its C-terminal region; this recruited frabin may deform the plasma membrane to induce outward membrane protrusion. Frabin activates Rac in the vicinity of the membrane structure(s), resulting in the WAVE-Arp2/3 system-induced generation of branched F-actin and WAVE-MDP system-induced outward membrane protrusion. The synergistic reorganization of the actin cytoskeleton and the plasma membrane finally induces the formation of lamellipodia. N, N-terminal region of frabin; DH-PH, the DH and first PH domains of frabin; C, C-terminal region of frabin; MDP, membrane-deforming protein.

Cell shape change has long been thought to be determined by the cytoskeleton reorganization beneath the plasma membrane. However, this idea could be revised by the discovery of several membrane-deforming proteins (MDPs), such as the BAR domain-containing endophilin and EFC domain-containing FBP17, which bind to WASP and WAVE proteins [3]. It is conceivable that cell shape changes are determined by the synergistic reorganization of the cytoskeleton and the plasma membrane. Therefore, we propose a model for the mode of action of frabin in the formation of filopo-dia and lamellipodia, as follows: initially, frabin is targeted to a preexisting specific actin structure through its FAB domain (Fig. 3). Once recruited, frabin reorganizes the actin cytoskeleton through the action of its N-terminal region, including the FAB and DH domains, in a Cdc42-independent manner. In addition, frabin activates Cdc42 through the DH domain and the first PH domain in the vicinity of the actin structure(s), resulting in the WASP/N-WASP-Arp2/3 system-induced generation of branched F-actin. The F-actin-bundling activity of frabin may contribute to the formation of bundled F-actin in filopodia. Furthermore, Cdc42 stimulates actin polymerization *via mDia2*. The cooperation of Cdc42-independent and Cdc42-dependent actin reorganization finally induces the formation of filopodia. This newly formed actin structure further recruits frabin in a positive feedback cycle to lengthen the filopodia.

Frabin is also recruited to a specific membrane structure(s) through the region including the DH domain, the first PH domain, the FYVE domain and the second PH domain [34]. Like BAR and EFC domains, this region may deform the plasma membrane to induce out ward membrane protrusions. Frabin activates Racinthe vicinity of the membrane structure(s), resulting in the WAVE-Arp2/3system-induced generation of branche dF-actin and WAVE-MDP system-induced outward membrane protrusion. The synergistic reorganization of the actin cytoskeleton and the plasma membrane finally induces the formation of lamellipodia.

## Mode of activation of frabin and FRG

It is unclear how external or internal stimuli transduce the signals to activate frabin and FGDs; but our recent preliminary results show that some phosphoinositides have a potency to stimulate the GEF activity of frabin. It has also been shown that frabin is recruited by the activation of phosphatidylinositol 3-kinase (PI3 kinase) during parasite invasion [39]. Frabin may be activated by 3-phos-phoinositides in the PI3 kinase signalling pathway.

In contrast to frabin and FGDs, the mode of activation of FRG is well studied. FRG is involved in the signalling pathway from the endothelin A receptor to JNK [14]. In this pathway, c-Src phos-phorylates FRG and activates Cdc42-specific GEF catalytic activity. The N-terminal region and the DH and PH domains are tyrosine-phosphorylated, but it remains unknown as to how this phospho-rylation leads to the activation of FRG. FRG is also involved in the signalling pathway underlying the cell adhesion-induced activation of Cdc42 [29, 32]. Cdc42 regulates the formation of cadherin-based adherens and claudin-based tight junctions through actin reorganization in epithelial cells [40, 41]. Nectin, a cell–cell adhesion molecule, recruits and activates c-Src at the nectin-based cell–cell contact sites, where FRG is then recruited, tyrosine-phos-phorylated by c-Src and activated. Rap1 small G protein is also recruited to the nectin-based cell–cell contact sites and locally activated there through the action of the c-Src-Crk-C3G signalling pathway. Activation of Rap1, in addition to the activation of c-Src, is required for the activation of FRG. The activation of either c-Src or Rap1 is insufficient for the activation of FRG. It has been shown that PI3 kinase functions downstream of Rap1 [42]; however, wortmannin, a PI3 kinase inhibitor, does not inhibit the nectin-induced activation of Cdc42, indicating that PI3 kinase is not involved in the activation of FRG [32, 43].

## Involvement of *FGD4/frabin* and *FGD1* in human diseases

Many pathogen microbes including viruses, bacteria and parasites utilize host cell actin for multiple actions such as attachment, entry into cells and movement within and between cells [44]. *Cryptosporodium parvum* (*C. parvum*), an intracellular parasite, is one of the most commonly reported enteric pathogens worldwide. Frabin was shown to mediate the cellular invasion of this parasite [39]. As described above, *C. parvum* recruits PI3 kinase to the host cell–parasite interface, an event that then results in the recruitment of frabin, leading to the activation of Cdc42 and subsequent host–cell actin reorganization during cellular invasion.

Recently, mutations in FGD4/frabin have been identified to be responsible for the Charcot-Marie-Tooth (CMT) disorder type 4H [24, 45]. CMT disorders are clinically and genetically heterogeneous hereditary motor and sensory neuropathies characterized by muscle weakness and wasting, foot and hand deformities and electrophysiological changes. The CMT4H subtype is an autosomal recessive demyelinating form of CMT disorder. Patients show early disease onset, but slow progressive sensori-motor neuropathy, and most of them present with severe scoliosis. The nerve biopsy specimens display a severe loss of myelinat-ed fibers, thinly myelinated axons and outfolding of myelin sheaths. These data indicate that frabin plays an important role in the proper myelination of the peripheral nervous system.

FGD1 was originally determined by positional cloning to be the gene responsible for an X-linked skeletal dysplasia, FGDY (also known as Aarskog-Scott syndrome). Mutations in the *FGD1* gene induce alternations in the size and shape of a number of small bones and cartilage elements [46]. The cardinal features of this disease include widely spaced eyes (hypertelorism), ptosis, down-slanting palpebral fissures, dysplastic ears, maxillary hypoplasia and disproportionate acromelic short stature. During embryogenesis, *FGD1* is initially expressed during the onset of ossification and its expression is limited to the ossifying skeletal components, including the cranio-facial bones [25]. This expression pattern directly corresponds to the abnormalities of bone formation observed in FGDY. It is proposed that FGD1 plays a role in ossification during skeletal formation.

## Conclusions and perspectives

We have described here our current knowledge of frabin and other related Cdc42-specific GEFs, including FGD1, FGD2, FGD3, FGD5, FGD6 and FRG. They all have multiple phosphoinositide-binding domains, including two PH domains and an FYVE or FERM domain. It is likely that they couple the actin cytoskeleton with the plasma membrane and regulate the membrane-dependent actin reorganization. Frabin associates with the specific actin and membrane structures and activates Cdc42 and Rac in the vicinity of these structures, resulting in the reorganization of the actin cytoskeleton and the plasma membrane. Thus, it is becoming clear how frabin induces cell shape changes. However, important questions still remain to be solved: (1) how external stimuli and intracellular signals induce the activation of frabin, (2) the physiological significance of the presence of frabin-β and -γ, (3) how frabin induces the activation of Rac and (4) how frabin regulates myelination. To address these issues, the FAB domain and a fragment containing the mutated DH domain, the first PH domain, the FYVE domain and the second PH domain may be useful because they have DN effects. Further studies are necessary for a better understanding of the modes of action and activation of frabin.
